# Carbapenem-resistant *Klebsiella pneumoniae* bloodstream infections in haematological malignances and hematopoietic stem cell transplantation: Clinical impact of combination therapy in a 10-year Brazilian cohort

**DOI:** 10.1371/journal.pone.0297161

**Published:** 2024-01-26

**Authors:** Ingvar Ludwig Augusto de Souza, Paola Cappellano, Diogo Boldim Ferreira, Maria Daniela Bergamasco, Thomas Cardoso das Chagas Neto, Fabio Rodrigues Kerbauy, Otavio Carvalho Guimarães Baiocchi, Antonio Carlos Campos Pignatari

**Affiliations:** 1 Disciplina de Infectologia, Departamento de Medicina, Escola Paulista de Medicina, Universidade Federal de São Paulo, Sao Paulo, Brazil; 2 Hcor–Hospital do Coracao, Sao Paulo, Brazil; 3 Fleury–Medicina e Saúde, Sao Paulo, Brazil; 4 Laboratório Central, Hospital São Paulo, Disciplina de Medicina Laboratorial, Departamento de Medicina, Escola Paulista de Medicina, Universidade Federal de São Paulo, Sao Paulo, Brazil; 5 Disciplina de Hematologia e Hemoterapia, Departamento de Oncologia Clínica e Experimental, Escola Paulista de Medicina, Universidade Federal de São Paulo, Sao Paulo, Brazil; Shiraz University of Medical Sciences, ISLAMIC REPUBLIC OF IRAN

## Abstract

Bacterial bloodstream infections (BSI) are a common threat among patients with haematological malignancies (HM) and hematopoietic stem cell transplant recipients (HSCT). The purpose of this research was to describe clinical and microbiological aspects of BSI caused by carbapenem-resistant *Klebsiella pneumoniae* (CRKp) and assess risk factors associated with 30-day mortality in a 10-year cohort of haematological patients. A total of 65 CRKp-BSI episodes occurring in HM patients and HSCT recipients and CRKp-BSI between January 2010 and December 2019 were retrospectively studied. Acute leukemias were the most frequently observed underlying disease (87.7%) and 18 patients (27.7%) received HSCT. Mucosal barrier injury in the gastrointestinal tract was the primary cause of bacteremia (86.1%). Also, 14 individuals (21.6%) had an Invasive Fungal Disease (IFD) throughout the episode. Regarding treatment, in 31 patients (47.7%) empirical therapy was deemed appropriate, whereas 33 (50.8%) patients received a combination therapy. Microbiological data revealed that the majority of isolates (53–58%) had the Polymyxin B co-resistance phenotype, while amikacin resistance was less common (16 samples, or 24.7%). The mortality rates at 14 and 30 days were 32.3% and 36.9%, respectively. In a multivariate Cox regression analysis, prompt appropriate antibiotic administration within three days was associated with a better outcome (Adjusted Hazard Ratio [aHR]: 0.33; 95% Confidence Interval [CI]: 0.14–0.76; p = 0.01), whereas hypotension at presentation (aHR: 3.88; 95% CI: 1.40–10.74; p = 0.01) and concurrent IFD (aHR: 2.97; 95% CI: 1.20–7.37; p = 0.02) were independently associated with death within 30 days. Additionally, a favorable correlation between combination therapy and overall survival was found (aHR: 0.18; 95%CI: 0.06–0.56; p = 0.002). In conclusion, 30-day mortality CRKp-BSI was elevated and most of the isolates were polymyxin B resistant. Early appropriate antimicrobial treatment and the use of combination therapy were linked to a better outcome.

## Introduction

Patients with haematological malignances (HM) undergoing high-intensity cytotoxic chemotherapy and hematopoietic stem cell transplantation (HSCT) recipients can be considered among the most severely immunosuppressed hosts. Neutropenia, the presence of central venous catheters, chemotherapy-induced mucosal lesions of the gastrointestinal tract, and graft-versus-host disease (GVHD) remain key factors in the complex combination of conditions contributing to the high incidence and prevalence of multiple and simultaneously infections in this scenario [1]. Bacterial bloodstream infections (BSI) continue to represent a significant burden in HSCT recipients and patients with HM. In this population, crude mortality rates can exceed 30% while bacteremia rates can surpass 40% [2–4].

*Klebsiella pneumoniae* is an established pathogen in healthcare associated infections, especially in cancer patients [5]. Moreover, it is a matter of concern that past two decades have been marked by the widespread emergence of carbapenem-resistant (CR) Gram-negative bacteria (GNB) on a global scale [6]. Within this context, Carbapenem-resistant *Klebsiella pneumoniae* (CRKp) emerged as a particularly significant challenge in certain regions, such as Brazil [7–10]. CRKp-BSI are associated with poor outcomes, elevated morbidity, mortality, and increased health costs, posing it as a major public health issue as well as a global economic threat [11–14]. Several risk factors for CRKp-BSI have been described, older age (over 60 years), need for intensive care treatment, immunosuppression, extended hospitalization, and exposure to broad-spectrum antimicrobials [15–18]. These conditions are commonly found amidst HM and HSCT patients, a population in which CRKp-BSI mortality could exceed 50% [19–22]. Despite its importance, CRKp-BSI has no established first-line treatment, and high rates of inappropriate empirical and definitive antimicrobial treatment have been reported [23–25]. Furthermore, there is a scarcity of evidence risk factors for mortality and treatment options for CRKp-BSI in this setting, with no randomized trials cementing a strongly recommended approach [25–27].

The purpose of this study was to describe the clinical and microbiological characteristics of CRKp-BSI in haematological patients and to determine the 30-day mortality risk factors in a 10-year single-center Brazilian cohort.

## Patients and methods

### Study design, population, setting and data collection

An observational, retrospective study was carried out at Hospital São Paulo, a 700-bed tertiary care facility and teaching hospital affiliated with the Universidade Federal de São Paulo, located in Sao Paulo, Brazil, which primarily serves patients from the Brazilian public health system and offers all medical specialties, with an emphasis on highly complex procedures.

This study included all consecutive episodes of CRKp-BSI diagnosed from adult patients (aged 17 years or older) with HM and/or HSCT recipients admitted from January 2010 to December 2019 who received at least one day of antimicrobial therapy following the collection of CRKp-positive blood cultures. Individuals who died before this period were not included. Only the first episode of each patient was included. A CRKp-BSI was diagnosed through the isolation of CRKp from blood cultures of patients exhibiting febrile neutropenia (FN) and/or symptoms of infection, such as hypotension and acute organ disfunction. The time when blood cultures were collected determined the onset of the episode.

Clinical data were collected from hospital charts and laboratory database before being entered into a study-specific electronic formulary, and included: demographics; comorbidities; baseline malignancy characteristics and performance status; healthcare exposures, such as anti-neoplastic chemotherapy administration (in the 30 days preceding CRKp-BSI) and the presence of a central venous catheter; donor selection, HSCT modality and conditioning regimen; previous (in the 30 days preceding CRKp-BSI) and concomitant infections, including invasive fungal disease (IFD); prior CRKp gut colonization (in the 180 days preceding CRKp-BSI); antibacterial, antifungal prophylaxis, antimicrobial therapies and exposure to carbapenems, polymyxin B and fourth-generation cephalosporin (in the 30 days preceding CRKp-BSI); neutropenia and severe neutropenia at the onset, throughout and at the end of a CRKp-BSI episode; hypotension on clinical presentation; Pittsburg bacteremia score (Pitt score) [28]; demand for intensive care unit (ICU); acute renal failure and need for renal replacement therapy; primary source of bacteremia; microbiological data, namely polymicrobial infection, etiologic pathogens and their susceptibility pattern; empirical and definitive antimicrobial treatment; time until receipt of an active drug; number of active drugs used in the treatment regimen; appropriateness of empirical and definitive therapy; use of combination therapy; underlying disease complications; 30-day mortality from CRKp-BSI onset and CRKp-BSI-related mortality (definitions below). Retrospective data collection and research processing occurred between November 2018 and June 2022.

All information was stored in computerized databases, ensuring privacy and anonymity of the patients enrolled.

This research was conducted with the approval of the local Ethics Committee (CEP—2.062.684/2017).

### Diagnostic and routine practices

Antibacterial prophylaxis with a fluoroquinolone (ciprofloxacin 500mg b.i.d. or levofloxacin 500mg q.d.) was routinely recommended during neutropenia in all acute leukemia and HSCT patients during the study period. Fluconazole (400mg q.d.) was used as primary anti-fungal prophylaxis during neutropenia, and serum galactomannan antigen test monitoring was used as part of the antifungal diagnostic-driven approach. Furthermore, patients with a prior history of filamentous IFD, as well as selected higher risk individuals undergoing allogeneic HSCT, were administered voriconazole (4 mg/kg q12h hours for intravenous administration, or 200 mg q12h when administered orally) for secondary or primary anti-mold prophylaxis. Blood cultures were obtained upon the presentation of fever or signs of infection, as determined by the attending physician’s clinical judgment. The selection of empirical antimicrobial therapy was guided by institutional protocols established by the Onco-Hematological Infections Study Group, which were based on local epidemiological and microbiological data. Typically, the first-line antimicrobial was an anti-pseudomonal β-lactam. Since 2012, the combination of a β-lactam with an aminoglycoside or polymyxin B was recommended as empirical therapy for high-risk patients. This recommendation was based on individual patient characteristics and predisposing factors for carbapenem-resistance (CR), as part of a de-escalation approach [29]. In addition, patients were screened weekly for CR gut colonization in accordance with institutional surveillance recommendations.

### Definitions

All *Klebsiella pneumoniae* isolates that exhibited in vitro resistance to at least one carbapenem (either ertapenem, imipenem, or meropenem) were collectively referred to as CRKp. The Eastern Cooperative Oncology Group (ECOG) scale was used to evaluate performance status [30]. IFD was classified according to the European Organization for Research and Treatment of Cancer/Mycosis Study Group (EORTC/MSG) [31]; only proven or probable IFD cases diagnosed or treated throughout the episode were included in the analysis. Prior CRKp gut colonization was defined as any CRKp cultured from stool or rectal swab samples obtained within the previous 180 days of the episode’s onset. Neutropenia and severe neutropenia were defined as absolute neutrophil counts of less than 500 cells/mm^3^ and 100 cells/mm^3^, respectively, whereas persistent neutropenia denoted presence of after the fourteenth day or at death. Hypotension on clinical presentation was characterized as having a systolic blood pressure less than 90 mmHg, or 40mmHg less than the patient’s baseline, within one day before or after the onset of CRKp-BSI. Acute renal failure was evaluated and classified during the infection duration based on the Acute Kidney Injury Network (AKIN) [32]. Definitions for health-care associated CRKp-BSI and infection sources have been previously brought forth [33]. Accordingly, mucosal barrier injury laboratory-confirmed bloodstream infection (MBI-LCBI) was diagnosed in patients whose blood cultures tested positive for *Klebsiella pneumoniae–*an organism included on the National Healthcare Safety Network NHSN MBI organism list–unrelated to an infection at another site, and who experienced neutropenia within a 7-day window encompassing the date when the positive blood culture was collected. Empirical therapy was considered appropriate if patients were administered at least one intravenous antimicrobial that showed fully in vitro activity against CRKp–hereafter referred to as CRKp active-drug–within 24 hours of blood cultures collection and prior to the availability of microbiological phenotype and susceptibility test results. Once the microbiological work-up was completed, definitive therapy was initiated. All GNB-spectrum antimicrobials used for more than one day were included. The use of a single CRKp active-drug in the “definitive therapy” regimen was considered monotherapy, while the simultaneously use of two or more CRKp active-drugs was categorized as combination therapy. Treatment was deemed inappropriate if it did not include a CRKp active-drug as part of the regimen. Underlying disease complications included events unrelated to the infection, such as hemorrhage, procedural setbacks, and major cardiovascular events such as arrhythmia, pulmonary embolism, or acute myocardial infarction. Thirty-day mortality accounted for deaths by any cause within the first 30 days of CRKp-BSI onset, while CRKp-BSI-related mortality specified deaths occurring within 14 days of infection.

### Microbiological workflow

Blood samples were cultured using Bactec^TM^ 9240 or Bactec^TM^ FX 40 systems (Becton Dickinson, Microbiology Systems, Cockeysville, MD, USA) and were incubated for a period of five days. Rectal swabs or stool samples were used to obtain surveillance cultures, which were then inoculated onto carbapenem-selective medium (MacConkey agar). Identification to the species level and susceptibility testing were carried out using the Phoenix^TM^ 100 automated system (Becton Dickinson, Microbiology Systems, Cockeysville, MD, USA) and/or by phenotypic standard procedures and disk-diffusion method. Results were interpreted according to Clinical and Laboratory Standards Institute (CLSI) criteria [34]. Minimum inhibitory concentrations (MICs) for polymyxin B were routinely determined by either broth microdilution (BMD) or gradient diffusion test (E-test®, bioMerieux, Marcy-L’Etoile, France). The interpretation of these breakpoints followed the guidelines of the European Committee on Antimicrobial Susceptibility Testing (EUCAST) and Brazilian Committee on Antimicrobial Susceptibility Testing (BrCAST) [35]. Considering the study’s duration and updates in polymyxin B MIC recommendations, polymyxin B MIC values initially obtained through gradient diffusion were retrospectively verified by cation-adjusted BMD (Sigma-Aldrich, Copenhagen, Denmark) in all available samples, to determine sensitivity or resistance according to EUCAST and BrCAST breakpoints [36]. Susceptibility tests for tigecycline, performed upon request and contingent on drug availability, involved obtaining MICs through either disk diffusion or gradient diffusion tests. These tests were also interpreted in line with EUCAST and BrCAST guidelines [35].

### Statistical analyses

Continuous variables were reported as medians with interquartile ranges, whereas categorical variables were presented as percentages of their absolute values. The Shapiro-Wilk test was used to determine if continuous variables had a normal distribution. Chi-square or Fisher’s exact tests were used for comparing categorical variables, and the Mann-Whitney or Student’s t-test were used for continuous variables. The statistical tests were conducted as two-tailed tests with a significance level of 0.05.

Survival analysis was performed comparing Kaplan-Meier curves using log-rank tests. Additionally, adjusted survival curves were generated to align with the results of the multivariate analysis. The adjusted hazard ratio (aHR) and its corresponding confidence intervals were obtained from the selected models.

A multivariate analysis using Cox proportional hazards regression was utilized to identify risk factors associated with 30-day survival. To address for confounders, all variables with p-values of 0.25 in bivariate analysis, as well as those with biological plausibility and substantiated in the literature, were manually aggregated in reverse stepwise fashion according to their biological relevance. Additionally, the analysis ensured the absence of collinearity and multicollinearity among the chosen variables and other possible confounders, as confirmed by variance inflation factor assessments. Given the small sample size, a parsimonious model that provided greater discriminatory predictive power and agreement was preferred. Final model selection was determined by the Akaike Agreement Index (AIC), which was calculated from the likelihood ratio test. Additionally, a second model was built to specifically address the impact of combination therapy. Rather than appropriateness of therapy or time up to receipt of an active drug, treatment was categorized as inappropriate therapy (reference), monotherapy, and combination therapy, in accordance with the definitions previously stated. This model incorporated all the same variables as the initial multivariate analysis.

All analyzes were performed using R Studio software (R Studio, PBC, Version 1.3.1093, “Apricot Nasturtium" for macOS).

## Results

### Demographic and clinical characteristics

During the study period, 65 patients met the inclusion criteria, resulting in 65 episodes of CRKp-BSI being included for analysis. Table 1 summarizes their demographic and clinical characteristics. The median age was 49 years, ranging from 17 to 83, with 20% of patients being over 65 years old. Overall, 89% of patients had an ECOG performance status ≤2 at baseline treatment. The most prevalent underlying diseases were acute leukemias (87.7%), with 41 episodes (63.1%) of Acute Myelogenous Leukemia (AML) and 16 individuals (24.6%) presenting with Acute Lymphoblastic Leukemia (ALL). Remission of HM at CRKp-BSI onset was observed in 18.5% of patients. There were 18 HSCT recipients, mostly allogeneic (72.2%). Furthermore, the majority of patients were neutropenic and/or severely neutropenic at the CRKp-BSI onset (93.8% and 87.7%, respectively).

**Table 1:** Clinical and demographical characteristics of patients.

Characteristics	Episodes, N = 65	(%)
**Demographics**		
Age–years, median (IQR)	49	(35.3–62.6)
Elderly (age > 65a)	13	(20)
Female sex	31	(47.7)
Male sex	34	(52.3)
**Comorbidities**	42	(64.6)
Hypertension	16	(24.6)
Diabetes mellitus	7	(10.8)
Cardiac failure	5	(7.8)
Smoking	18	(27.7)
**Underlying disease**		
Acute myelogenous leukemia	41	(63.1)
Acute lymphoblastic leukemia	16	(24.6)
Lymphomas	4	(6.1)
Other myeloproliferative or lymphoproliferative disorders	4	(6.1)
**Status of haematological malignance**		
Newly diagnosed–first line treatment	24	(36.9)
Refractory	16	(24.6)
Relapsed	13	(20)
Remission	12	(18.5)
**Hematopoietic stem cell transplantation**	**18**	**(27.7)**
• Allogenic: HLA compatible sibling	5	(7.7)
• Allogenic: matched-unrelated donor	4	(6.2)
• Allogenic: haploidentical	4	(7.7)
• Autologous	5	(6.2)
**Conditioning regimens** ^a^		
Reduced intensity conditioning regimen	6	(33.3)
Myeloablative	3	(16.7)
Non-myeloablative	9	(50)
**Clinical characteristics and exposures**		
ECOG > 2	7	(10.8%)
Chemotherapy^b^	59	(90.8)
Central venous line^c^	59	(90.8)
Antifungal prophylaxis^b^	65	(100)
Anti-mold prophylaxis^b^	12	(18.5)
Antibacterial prophylaxis–quinolone^b^	55	(84.6)
Previous therapeutic antimicrobial therapy^b^	60	(92.3)
Fourth generation cephalosporins exposure^b^	38	(58.5)
Carbapenems exposure^b^	49	(75.4)
Polymyxin B exposure^b^	11	(16.9)
Neutropenia^d^	61	(93.8)
Severe neutropenia^e^	57	(87.7)

Clinical and demographical characteristics of 65 carbapenem-resistant *Klebsiella pneumoniae* bloodstream infections among patients with haematological malignances and hematopoietic stem cell transplant recipients. Notes: HLA–Human Leucocyte Antigen; IQR–Interquartile range.

^a^considering only hematopoietic stem cell transplant recipients

^b^In the 30 days preceding CRKp-BSI

^c^At the CRKp-BSI onset

^d^Neutrophil count below 500 cel./mm^3^

^e^Neutrophil count below 100 cel./mm^3^

### Microbiological characteristics, clinical presentation, treatment and outcomes

In terms of microbiological results, 19 patients (28.4%) had a previous episode of BSI caused by another pathogenic micro-organism, and 14 individuals (21.6%) were diagnosed with/or treated for a IFD classified as probable or proven during the CRKp bacteremia. The most frequent IFD was aspergillosis, accounting for 10 cases (58.8% of fungal infections), followed by fungemia (hematogenous candidiasis, fusariosis and trichosporonosis). CRKp colonization was present in 19 patients (29.2%), with a higher frequency observed among HSCT recipients compared to non-HSCT recipients (55.6% vs. 18.3%; p = 0.005). Mucosal barrier injury of the gastrointestinal tract was the most common source of bacteremia (86.1%), and six subjects (9.2%) experienced polymicrobial BSI, as shown in Table 2.

**Table 2:** Microbiological and clinical characteristics, presentation, treatment features and outcome.

Characteristics	Episodes, N = 65	(%)
**Microbiological features**		
Previous treated BSI^a^	17	(26.2)
Invasive fungal infection^b^	14	(21.6)
Gut colonization by CRKp^c^ (last 180 days)	19	(29.2)
**Source of bacteremia**		
Mucosal barrier injury of the gastrointestinal tract	56	(86.1)
Primary central venous catheter bloodstream infection	6	(9.2)
Skin and soft tissues infection	3	(4.7)
**Polymicrobial infection** ^**d**^	**6**	**(9.2)**
• *Acinetobacter baumanni*	1	(16.7)
• *Enterococcus faecalis*	1	(16.7)
• *Enterococcus faecium*	1	(16.7)
• *Fusarium solani*	1	(16.7)
• *Rothia mucilaginosa*	1	(16.7)
• *Streptococcus parasanguinis*		
Polymyxin B resistant CRKp^e^	34	(52.3)
**Clinical presentation**		
Hypotension on presentation	26	(40)
Pitt Score ≥ 4	10	(15.4)
Demand for intensive care unit	25	(38.5)
Acute renal failure	43	(66.2)
Acute renal failure (AKIN II or III)	27	(41.5)
**Treatment attributes**		
Appropriate empirical therapy	31	(47.7)
Appropriate therapy within 3 days	47	(72.3)
Appropriate definitive therapy	53	(81.5)
CRKp combination therapy	33	(50.8)
Number of CRKp-active drugs used in treatment		
• 0	12	(18.4)
• 1	20	(30.8)
• ≥ 2	33	(50.8)
**Outcomes**		
Underlying disease complications	11	(16.9)
14-day mortality	21	(32.3)
30-day mortality	24	(36.9)

Microbiological and clinical characteristics, presentation, treatment features and outcome of 65 carbapenem-resistant *Klebsiella pneumoniae* bloodstream infections among patients with haematological malignances and hematopoietic stem cell transplant recipients. Notes: AKIN–Acute kidney injury network; BSI–Bloodstream infection; CRKp–Carbapenem-resistant *Klebsiella pneumoniae*; ECOG–Eastern Cooperative Oncology Group; MIC–minimum inhibitory concentration.

^a^In the 30 days preceding CRKp-BSI

^b^Only probable or proven diagnosed or treated during the episode

^c^In the 180 days preceding CRKp-BSI

^d^Proportionally to the number of polymicrobial infections

^e^Polymyxin B MIC > 2mcg/mL

**Fig 1:**
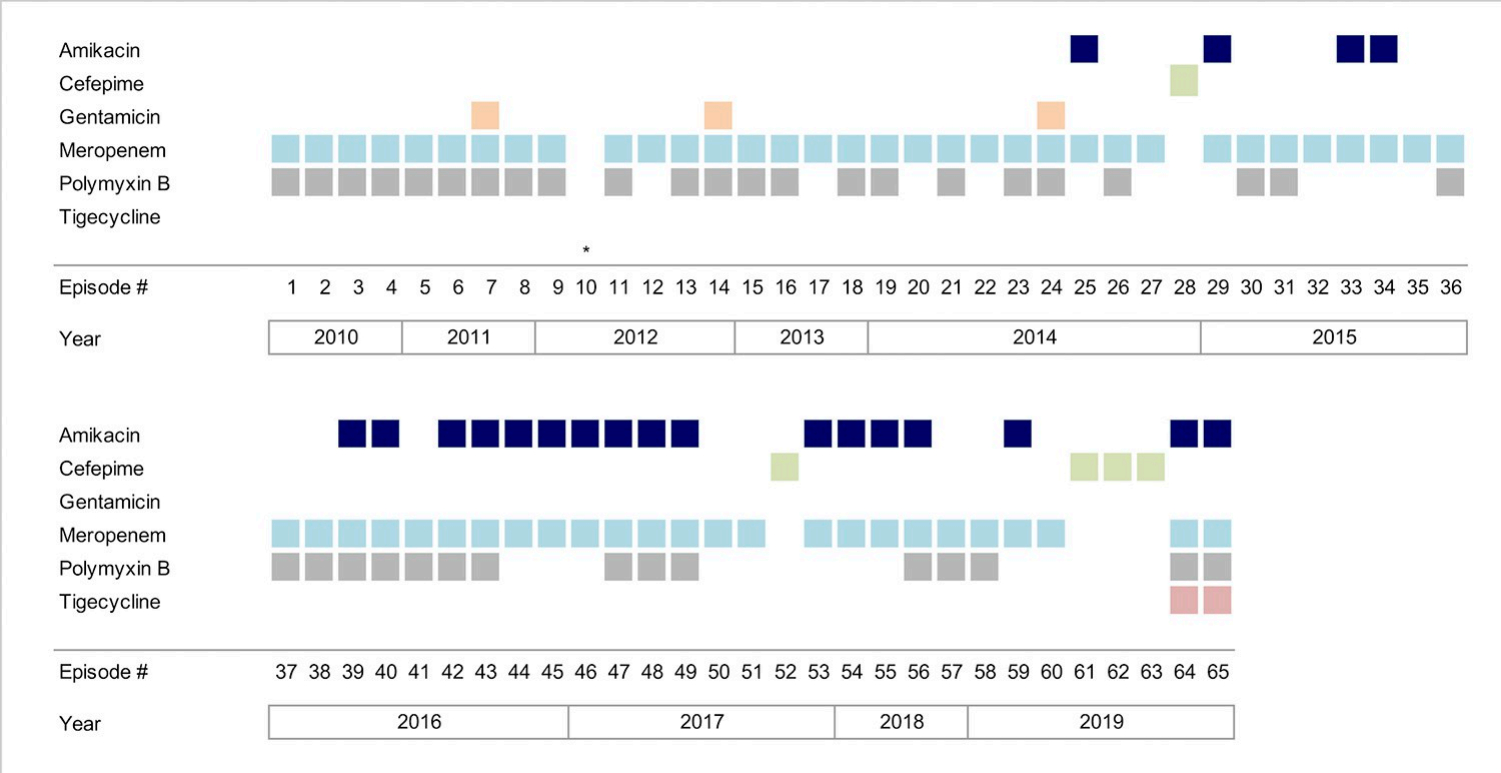
Graphical timeline: Gram-negative spectrum antimicrobials used as empirical treatment. The figure illustrates the empirical treatment choices made in the 65 episodes of CRKp-BSI over the 10-year study period, highlighting the dynamic and diverse nature of therapeutic decisions. Each row represents an antimicrobial used as empirical treatment given the episode’s number, yearly stratified (columns). *episode #10 did not received empirical treatment since it was non-neutropenic or had any signs of organ disfunction; antimicrobial therapy was started right after the outcome of initial microbiological tests.

Sepsis with cardiovascular dysfunction (hypotension) was recorded in 26 patients (40%), and 10 (15.4%) had a Pitt score ≥ 4 points (median: 1; interquartile range: 0–2). In 31 episodes (47.7%) the empirical therapy was considered appropriate, and the empirical antimicrobial regimens are illustrated in Fig 1. In total, 18 different treatments were used. Definitive antimicrobial therapy was appropriate in 81.5% of patients, and combination therapy was used in 50.8% of subjects. However, almost 20% of individuals did not receive any CRKp-active drug. Furthermore, a considerable diversity in therapeutic regimens was observed across all three treatment groups: non-CRKp active drug, monotherapy, and combination therapy, as illustrated in supporting information Fig 1 (S1 Fig). CRKp-BSI-related and 30-day all-cause mortality rates were 32.3% and 36.9%, respectively (Table 2).

### Microbiological characteristics of CRKp isolates

**Fig 2:**
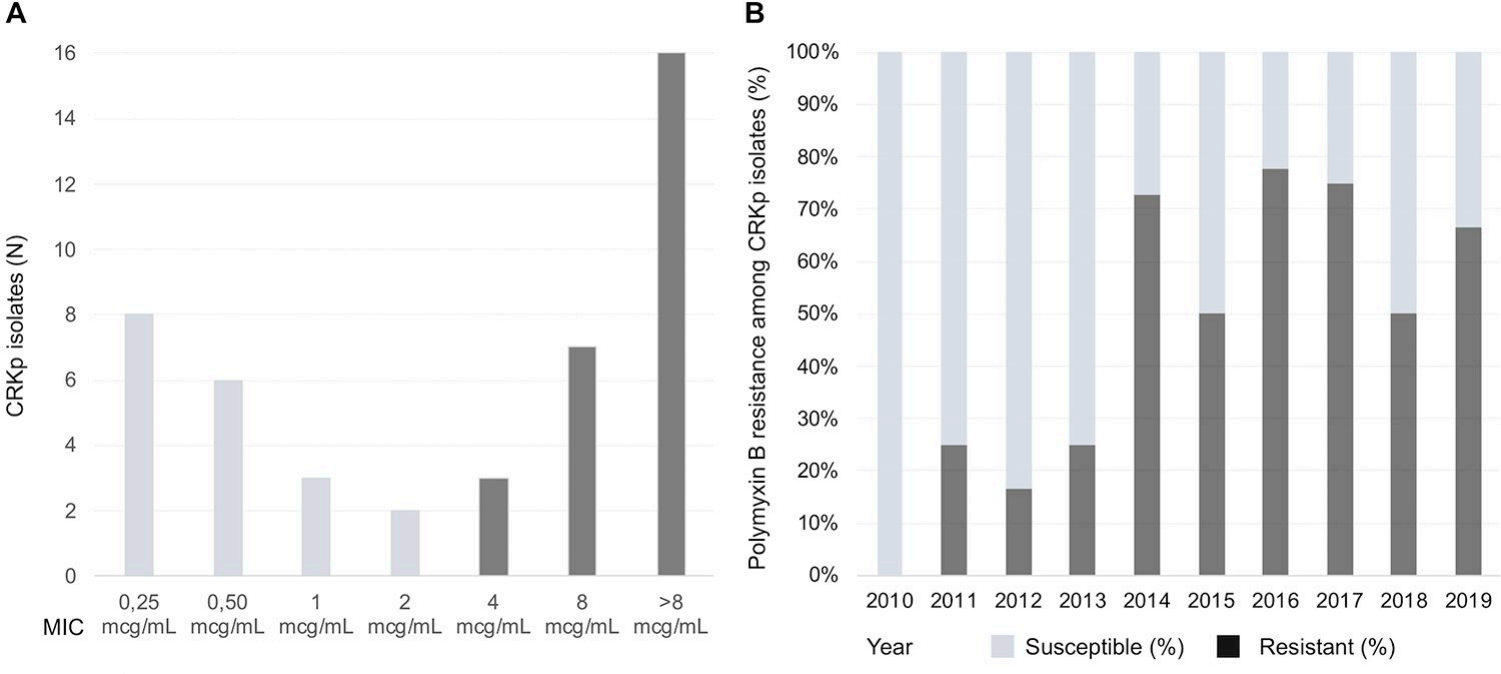
MIC distribution and polymyxin B resistance in carbapenem-resistant *Klebsiella pneumoniae*, January 2010—December 2019. Minimum inhibitory concentrations distribution and proportion of polymyxin B resistance among carbapenem-resistant *Klebsiella pneumoniae* isolates from January 2010 to December 2019. (A) Absolute number of samples distributed according to the minimum inhibitory concentrations obtained by broth microdilution of 45 carbapenem-resistant *Klebsiella pneumoniae* isolates. Susceptibility (light grey), resistance (dark grey). (B) Percentages of susceptible (light grey) and resistant (dark grey) polymyxin B isolates amidst 65 episodes of carbapenem-resistant *Klebsiella pneumoniae* bloodstream infections, yearly stratified. Notes: CRKp—carbapenem-resistant *Klebsiella pneumonia*; MIC–Minimum inhibitory concentration.

Isolates’ susceptibility profile demonstrated an elevated prevalence of antimicrobial resistance among different classes. Amikacin demonstrated the highest susceptibility rate, with only 16 strains (24.6%) showing in vitro resistance. Gentamicin, another aminoglycoside tested, was found to be resistant in 49 out of the 65 isolates (75%). Additionally, there was a notable increase in gentamicin resistance starting from 2013, with resistance being observed in over 85% of the samples. Carbapenems also displayed a high level of resistance: 85% of isolates had a MIC for meropenem equal to or greater than 16mcg/mL. All strains were resistant to quinolones (Table 3). Polymyxin B resistance was observed in 34 out of the 65 samples (53.2%). Among those with MIC confirmed by BMD, 26 out of the 45 strains (58%) had a MIC > 2 mcg/mL (Fig 2A). The first detection of this phenotype occurred in the second year of the study. In 2014, a notable surge was observed in the number of strains exhibiting co-resistance to carbapenems and polymyxin B. Specifically, eight out of the 11 isolates tested (approximately 73%) displayed this resistance profile in that year. After 2014, polymyxin B resistance remained frequent (Fig 2B). Susceptibility testing for tigecycline was conditioned on its clinical use, and a total of 27 isolates were tested, with resistance observed in 9 (33%) of them.

**Table 3:** Antimicrobial susceptibility of *Klebsiella pneumoniae* isolates.

Antimicrobial	Interpretative Resistance MIC Breakpoint, (mcg/mL)		MIC (mcg/mL)		Resistant Isolates, N	(%)
		Minimum	Maximum	Median (*p*_50_)		
Amikacin	≥ 64	< 8	≥ 32	< 8	16	(24.6)
Gentamicin	≥ 16	< 2	≥ 8	≥ 8	49	(75.3)
Ciprofloxacin	≥ 4	2	≥ 2	≥ 2	65	(100)
Cefepime	≥ 16	16	≥ 16	≥ 16	65	(100)
Imipenem	≥ 4	0.25	≥ 32	≥ 8	60	(92.3)
Meropenem	≥ 4	0.25	≥ 32	≥ 8	62	(95.4)
Polymyxin B	> 2	0.125	64	> 2	34	(52.3)
Tigecycline^a^	> 1	0.5	> 4	1	9	(33)

Antimicrobial susceptibility of 65 carbapenem-resistant *Klebsiella pneumoniae* bloodstream isolates. Notes: MIC–Minimum inhibitory concentration; *p*_50_ – 50^th^ percentile of isolates

^a^Susceptibility tests for tigecycline were performed upon request and subject to drug availability. A total of 27 isolates were tested, with resistance observed in 9 (33%) of them.

### Risk factors for CRKp-BSI 30-day mortality

Table 4 summarizes the results of bi-variate analysis conducted to assess risk factors associated with death within 30 days. Hypotension on presentation (67% vs. 24%; p = 0.001), need for ICU care (67% vs. 22%; p < 0.001), and acute kidney failure during the episode (92% vs. 55%; p = 0.001) were more frequently observed among patients who died. Persistent neutropenia also differed significantly between the two groups. Of the 41 survivors, 11 individuals remained neutropenic on the fourteenth day. In contrast, 22 of the 24 deaths occurred during the course of neutropenia (26.8% vs. 91.6%; p < 0.0001). This variable had the strongest association with death, with a relative risk of 10.6 (95%CI: 2.73–41.7). Appropriate treatment and CRKp combination therapy were more prevalent amidst survivors.

**Table 4:** Bivariate analysis of predisposing factors associated with increased risk of death within 30 days.

Clinical, microbiological and therapeutic characteristics	Survivors, N = 41	(%)	Deaths, N = 24	(%)	RR	(CI 95%)	*p*
Age–year, median (IQR)	53.5	(34–63)	55.2	(42–63)	NA	NA	0.5
Elderly (age > 65-yo)	8	(20)	5	(21)	1.05	(0.48–2.28)	1
Male sex	22	(54)	12	(50)	0.91	(0.48–1.27)	0.8
Comorbidities	25	(61)	17	(71)	1.32	(0.62–2.73)	0.6
ECOG > 2	3	(7)	4	(17)	1.65	(0.79–3.44)	0.41
**Underlying disease**							
• Acute myelogenous leukemia	25	(61)	16	(67)	2.08	(0.7–6.2)	.
• Acute lymphoblastic leukemia	13	(32)	3	(13)	ref.	NA	0.11
• Other HM	3	(7)	5	(21)	3.33	(1.05–10.5)	.
Remission of underlying disease	10	(24)	2	(8)	0.4	(0.11–1.48)	0.18
Chemotherapy^a^	40	(98)	19	(79)	0.38	(0.23–0.84)	0.02
HSCT recipient	11	(27)	7	(29)	1.07	(0.53–2.14)	1
**Clinical characteristics and interventions**							
Central venous line	37	(90)	20	(92)	1.18	(0.35–3.64)	1
Neutropenia^b^	37	(90)	23	(96)	1.91	(0.32–11.4)	0.64
Severe neutropenia^c^	36	(88)	21	(88)	0.98	(0.37–2.55)	1
Persistant neutropenia^d^	11	(27)	22	(22)	10.6	(2.73–41.7)	< 0.0001
Underlying disease complications	4	(10)	7	(29)	2.02	(1.11–3.66)	0.08
**Microbiological features**							
Previous treated BSI^a^	11	(27)	6	(25)	0.94	(0.45–1.97)	1
Invasive fungal infection^e^	6	(15)	8	(33)	1.82	(0.99–3.34)	0.11
Gut colonization by CRKp^f^	14	(34)	5	(21)	0.63	(0.27–1.45)	0.39
Polymicrobial infection	5	(12)	1	(4)	0.42	(0.07–2.63)	0.4
Polymyxin B resistant CRKp^g^	18	(44)	16	(67)	1.82	(0.91–3.65)	0.12
**Clinical presentation**							
Hypotension on presentation	10	(24)	16	(67)	3.0	(1.5–5.97)	0.001
Pitt Score ≥ 4	2	(5)	8	(33)	2.75	(1.64–4.6)	0.003
Demand for intensive care unit	9	(22)	16	(67)	3.2	(1.61–3.65)	< 0.001
Acute renal failure	21	(51)	22	(92)	5.62	(1.45–21.78)	0.001
Acute renal failure (AKIN II / III)	12	(29)	15	(63)	2.34	(1.2–4.5)	0.01
**Treatment attributes**							
Appropriate empirical therapy	28	(68)	11	(46)	0.56	(0.3–1.06)	0.07
Appropriate therapy within 3 days	34	(83)	13	(54)	0.45	(0.25–0.81)	0.02
Appropriate definitive therapy	37	(90)	16	(67)	0.36	(0.15–0.84)	0.03
CRKp combination therapy	27	(66)	6	(25)	0.32	(0.15–0.71)	0.002

Bivariate analysis of predisposing factors associated with increased risk of death within 30 days in 65 patients with carbapenem-resistant *Klebsiella pneumoniae* bloodstream infection in patients with haematological malignances and hematopoietic stem cell transplant recipients. Notes: AKIN–Acute kidney injury network; BSI–Bloodstream infection; CRKP–Carbapenem-resistant *Klebsiella pneumoniae*; HM–Haematological malignances; HSCT–Hematopoietic stem cell transplant; IQR–Interquartile range; MIC–minimum inhibitory concentration.

^a^In the 30 days preceding CRKp-BSI

^b^Neutrophil count below 500 cel./mm^3^

^c^Neutrophil count below 100 cel./mm^3^

^d^Neutropenia after the fourteenth day or at death

^e^Only probable or proven diagnosed or treated during the episode

^f^In the 180 days preceding CRKp-BSI

^g^Polymyxin B MIC > 2mcg/mL

From the exploratory data analysis, and based on the aforementioned criteria, Cox proportional hazards regression models were elaborated to sought risk factors independently associated with 30-day survival, as show in Table 5.

**Table 5:** Factors associated with 30-day survival according to multivariate Cox regression model.

Clinical, microbiological and therapeutic characteristics	Crude	analysis	*p*	Adjusted	analysis	*p*
**Clinical exposures**	**HR**	**(CI 95%)**		**aHR**	**(CI 95%)**	
ECOG > 2	2.36	(0.79–7.1)	0.18	…	…	…
**Underlying disease**						
• Acute myelogenous leukemia	2.51	(0.73–8.64)	0.1	…	…	…
• Acute lymphoblastic leukemia	reference	…	…	…	…	…
• Other HM	4.64	(1.1–19.5)	0.03	…	…	…
Remission of underlying disease	0.34	(0.08–1.43)	0.14	…	…	…
Chemotherapy^a^	0.21	(0.08–0.57)	0.002	…	…	…
Persistant neutropenia^b^	16.72	(3.9–71.3)	< 0.001	…	…	…
Underlying disease complications	2.19	(0.91–5.28)	0.08	…	…	…
**Microbiological features**						
Invasive fungal infection^c^	2.16	(0.03–5.06)	0.07	2.97	(1.2–7.4)	0.02
Gut colonization by CRKp^d^	0.59	(0.22–1.58)	0.08	…	…	…
Polymyxin B resistant CRKp^e^	2.29	(0.97–5.36)	0.06	…	…	…
**Clinical presentation**						
Hypotension on presentation	4.9	(2.1–11.5)	< 0.001	3.88	(1.4–10.7)	0.01
Pitt Score ≥ 4	5.8	(2.45–13.71)	< 0.001	…	…	…
Demand for intensive care unit	4.68	(1.99–11.03)	< 0.001	2.16	(0.8–5.9)	0.13
Acute renal failure	7.12	(1.68–30.36)	0.008	…	…	…
Acute renal failure (AKIN II / III)	2.99	(1.3–6.9)	0.01	…	…	…
**Treatment attributes**						
Appropriate empirical therapy	0.47	(0.21–1.06)	0.07	…	…	…
Appropriate therapy within 3 days	0.34	(0.15–0.76)	0.009	0.33	(0.14–0.76)	0.02
Appropriate definitive therapy	0.29	(0.13–0.69)	0.005	…	…	…

Multivariate analysis of 30-day survival-modifying factors in 65 patients with carbapenem-resistant Klebsiella pneumoniae bloodstream infection, according to Cox proportional hazards model. All variance inflation factor values of the variables included in the final multivariate model were less than 1.5. Notes: AKIN–Acute kidney injury network; BSI–Bloodstream infection; CRKP–Carbapenem-resistant *Klebsiella pneumoniae;* ECOG– Eastern Cooperative Oncology Group; HM–Haematological malignances; HSCT: Hematopoietic stem cell transplant; MIC–minimum inhibitory concentration.

^a^In the 30 days preceding CRKp-BSI

^b^Neutropenia after the fourteenth day or at death

^c^Only probable or proven diagnosed or treated during the episode

^d^In the 180 days preceding CRKp-BSI

^f^Polymyxin B MIC > 2mcg/mL

**Fig 3:**
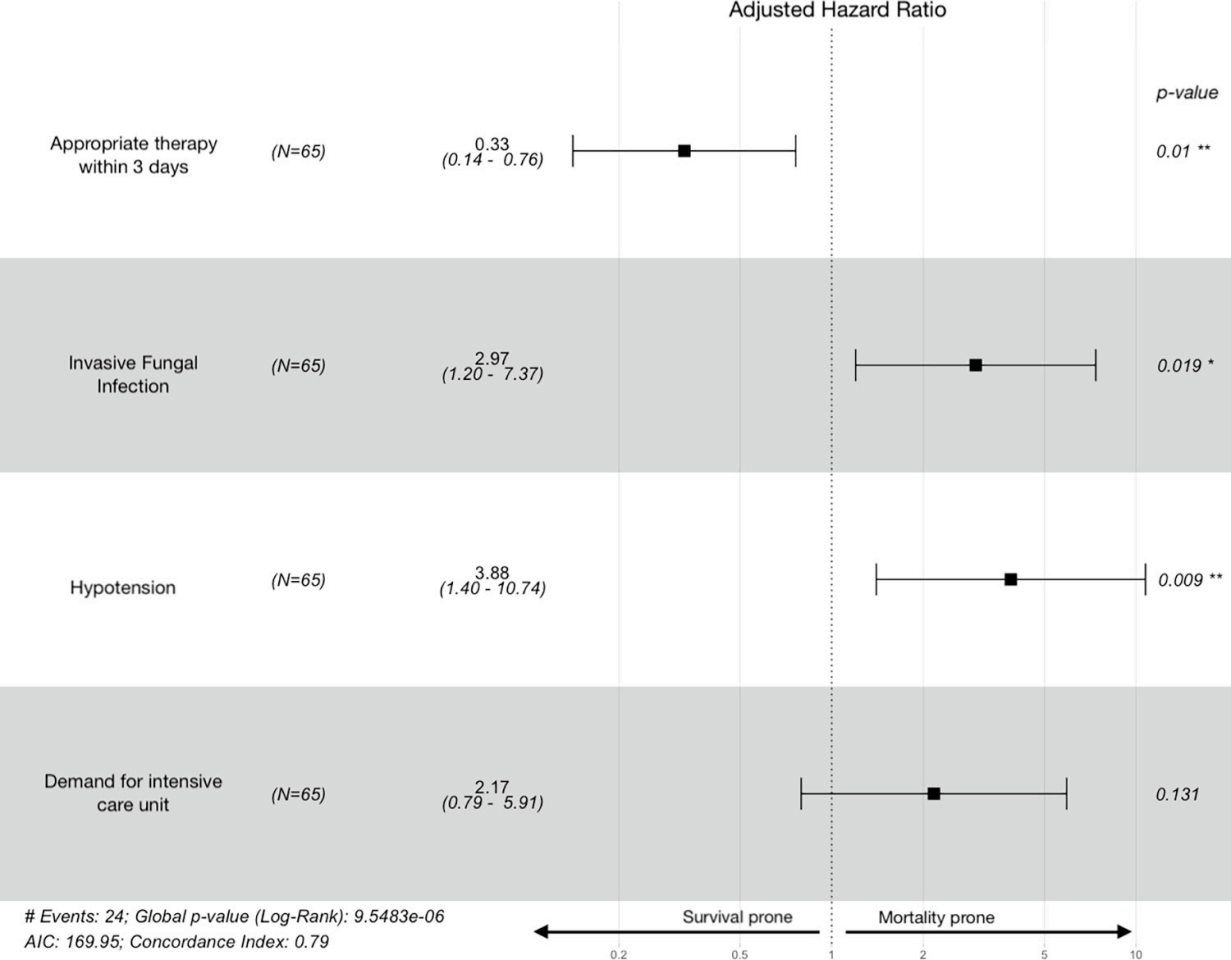
Forest plot of 30-day survival modifiers in carbapenem-resistant *Klebsiella pneumoniae* bloodstream infections. Attributable risk factors for 30-day mortality in 65 episodes of CRKp-BSI infection, according to the best fitted model. Hazard ratios (and its 95% confidence interval) values below one denotes a positive correlation with survival whereas values above are associated with mortality. Notes: AIC–Akaike information criteria. All variance inflation factor values of those variables included in the final multivariate model were less than 1.5.

Hypotension on presentation (aHR: 3.88; 95%CI:1.40–10.74; *p* = 0.01) and concomitant IFD (aHR: 2.97; 95%CI: 1.20–7.37; *p* = 0.02) were independently associated with death, whereas appropriate antimicrobial therapy initiated within 3 days (aHR: 0.33; 95%CI: 0.14–0.76; *p* = 0.01) of CRKp-BSI onset was correlated with a better outcome (Fig 3).

**Fig 4:**
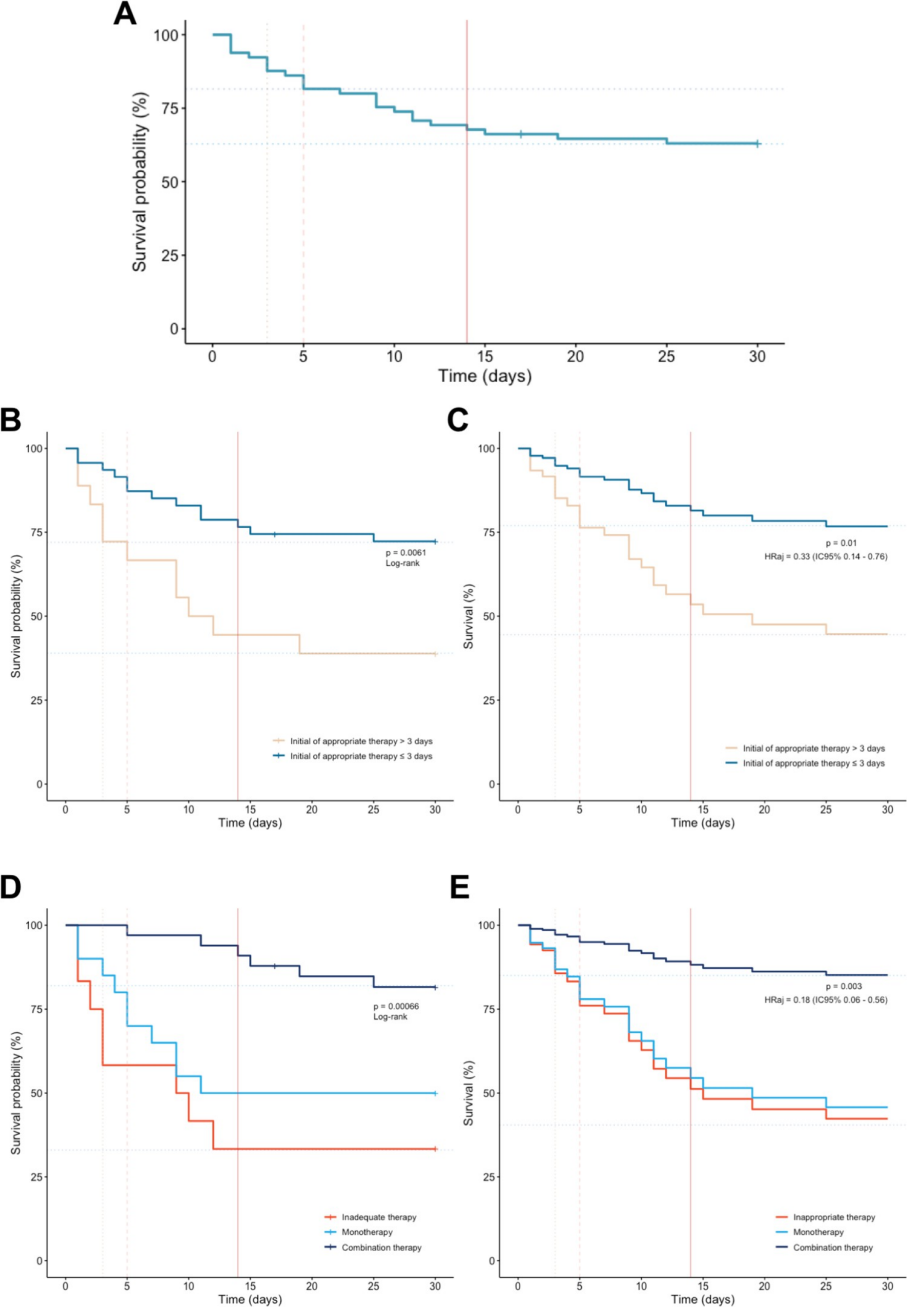
30-day Survival curves of all carbapenem-resistant *Klebsiella pneumoniae* bloodstream infection episodes. (A) Crude 30-day survival curve. (B) Comparative survival curves of individuals receiving appropriate antimicrobial therapy within 3 days and those who did not. (C) Survival curves for appropriate antimicrobial therapy within 3 days adjusted in concordance with the results obtained through the multivariate analysis (correct by hypotension, IFD and demand for ICU treatment). (D) Survival curves as per treatment classification: inappropriate therapy, monotherapy and combination therapy. (E) Survival curves as per treatment classification adjusted in concordance with the results obtained through the multivariate analysis (corrected by hypotension, IFD and demand for ICU treatment). Notes: Statistical significance was tested using the log-rank test, and P values are shown. The adjusted hazard ratio and its respective confidence intervals were retrieved from the selected model. All variance inflation factor values of the variables included in the final multivariate model were less than 1.5.

In the second model, which evaluated the impact of treatment modalities on survival, combination therapy was associated with a significant reduction in mortality risk (aHR: 0.18; 95%CI: 0.06–0.56; *p* = 0.003), which has been not observed with monotherapy (aHR: 0.86; 95%CI: 0.34–2.22; *p* = 0.76). Lastly, 30-day mortality among patients receiving inappropriate therapy, monotherapy and combination therapy were 66.6%, 50% and 18% (Fig 4).

### Survival analysis

Analysis of Kaplan–Meier overall survival curve reveals a sharp drop in the first two weeks of follow-up, when it tends to stability maintained until the thirtieth day (Fig 4). Further examination of the survival curves, illustrates the protective effect on mortality promoted by appropriate antimicrobial therapy initiated within 3 days, with an absolute risk reduction (ARR) of death of 33.4% (CI 95%: 7.5%–59%). Combination therapy was also correlated with lower mortality at 30-day (ARR: 37.9%; CI 95%: 16.1–59.7) as illustrated in Fig 4.

## Discussion

This study provides important insights into the characteristics and outcomes of CRKp-BSI in haematological patients and HSCT recipients. AML was the most common underlying disease among the patients (63.1%), consistent with findings from other studies [19, 20, 37]. As expected, previous antimicrobial exposure was also frequently observed in this analysis (92.3%). Concomitant IFD was found to be fairly prevalent in this cohort, affecting 21.5% of patients. Given that a majority of the patients had AML, high-risk condition for both bacterial and fungal infections, this finding is consistent with previously recent data from a prospective multicenter Brazilian study that evaluated the epidemiology of IFD and found that 26.1% of individuals with AML also had IFD [38]. It is important to note that haematological patients present a flawed infection model because they share several characteristics that increase the risk of concurrent and multiple infections, such as CRKp-BSI and IFD. Despite the prevalence and clinical significance, there is limited detailed information available regarding their coexistence and outcomes.

According to the proposed definitions [33], gastrointestinal mucosal barrier injury was considered the source of bacteremia in 86.1% of CRKp-BSI episodes in this study. This finding underscores the ongoing debate over routine surveillance for carbapenem-resistant (CR) colonization and the use of gut decontamination strategies [21]. Recently, this issue has gained more attention, emphasizing the need for appropriate methodological studies to determine the safety, benefits, and impact of CR-GNB decolonization in onco-hematological patients [21, 39]. In this cohort, previous CRKp gut colonization was recorded in 29.2% of individuals, with HSCT recipients having a higher frequency (55.6% vs. 18.3%; p = 0.005). Although frequently observed, this cannot be conclusively linked as a risk for CRKp-BSI. However, previous researches have established a correlation between CRKp gut colonization a heightened risk of subsequent CRKp bloodstream infections (BSI), as well as adverse long-term outcomes, particularly in hematopoietic stem cell transplant (HSCT) recipients. [40, 41].

The severity and impact of CRKp-BSI can be illustrated by the high proportion of patients with sepsis and acute organ dysfunction, namely hypotension (40%), demand for ICU treatment (38.5%), and acute kidney failure (66.2%). Refractory hypotension is linked to bacteremia and early death in onco-hematological patients during FN [42]. Apart from its severity, CRKp-BSI treatment is made difficult by several issues: CRKp frequently exhibits resistance to various antimicrobial classes; a lack of therapeutic arsenal and the toxicity of the remaining CRKp-active drugs; high rates of inappropriate empirical and definitive treatment; persistent neutropenia and immune deregulation; and potential concomitant infections (as previously stated) [43].

Despite an empirical CR-based strategy being formally adopted throughout most of the study, more than half of patients (52.3%) received inadequate empirical therapy, in accordance with recent studies [24, 25]. Moreover, multiple different definitive antimicrobial regimens were used to treat CRKp-BSI and carbapenems remained a partially component of the definitive treatment for the majority of patients, demonstrating the complexity of its therapeutic management (S1 Fig). A recent systematic review aimed at evaluating the role of combination therapy in severe infections caused by CR-GNB found a high heterogeneity of treatments, precluding any conclusion toward a better approach [44]. CRKp is now endemic in Brazil, where it is a public health concern. *Klebsiella pneumoniae* is the most common GNB pathogen in healthcare-associated BSI, with the majority (51.8%) being CR [45]. It is concerning that the majority of isolates in this cohort were also resistant to polymyxin B and most available antimicrobials.

Roughly 58% of the samples showed polymyxin B co-resistance, outnumbering previous reports [19, 20, 46]. Specifically, among those 34 patients, 12 did not receive any CRKp-active drug, thirteen patients were treated with monotherapy, often an aminoglycoside, while nine received combination therapy involving more than one drug with full in vitro activity. In this specific group, 30-day mortality was 47%, while overall 30-day mortality was 36.9%. This emphasizes the urgent need for new effective treatment strategies and the importance of strict antibiotic stewardship programs to prevent further emergence and spread of CRKp. In light of these observations, the resistance to quinolones, which was observed in all isolates, prompted a re-evaluation of its utility and role in the selection of CRKp strains. These findings resonate with previous data [47] and played a role in influencing the decision to discontinue the practice of universal quinolone prophylaxis during neutropenia in high-risk HM patients and HSCT recipients at our center. Polymyxin B has been institutionally adopted as empirical therapy in febrile neutropenia (FN) as part of a de-escalation approach strategy, tailored according to risk factors for CRKp. However, an increase in polymyxin B-resistant CRKp phenotypes has been noted since 2014, which is consistent with other studies [48, 49]. It is plausible to consider that the expanded use of polymyxin B may have contributed to this trend at our setting, which have prompted a reassessment of the use of polymyxin B as empirical therapy. Consequently, its application has now become more restricted, primarily reserved for patients with previous CRKp infections and/or gut colonization and in cases where superior empirical alternatives are absent.

Survival analysis revealed that 87.5% of deaths occurred within 14 days of CRKp-BSI onset. During this period, appropriate antimicrobial therapy is one of few modifiable factors and, indeed, receiving an appropriate antimicrobial therapy within 3 days had a protective effect on mortality in this cohort. Early appropriate treatment was associated with better outcomes in this multivariate analysis and should be pursued. Nonetheless, in the era of antimicrobial resistance, the use of an appropriate antimicrobial therapy prompted initiated in an episode of CRKp-BSI has proven to be a difficult achievement. A broad empirical therapy is frequently used, which is a well-established approach in febrile neutropenia in haematological patients. In 1972, Tattersal *et al*. proposed the use of five antimicrobials as the initial empirical treatment of FN, with polymyxin B and gentamicin constituting the majority of regimens [50]. Half a century later, the combination of amikacin and polymyxin B emerged as the most prevalent therapy in this cohort. While progress may encounter setbacks, combination therapy was independently linked to more favorable outcomes in the multivariate analysis, indicating that it might be the most advantageous strategy in these circumstances. An extensive variety in therapeutic regimens was discerned among all patients, highlighting the complexity and individualized nature of the treatment approaches. Aside from therapeutic decisions, the upcoming of faster microbiological diagnostic tools represents a promising approach, allowing prompt therapeutical adjustments and treatment optimization [51].

In a multivariate analysis, hypotension at presentation and IFD were linked to death. Hypotension is a well-known indicator of a poor outcome. Because of its importance, it is included in a variety of severity scores [52]. Patients with HM and HSCT recipients have such critical aspects that an isolated clinical data point, such as hypotension, can identify those who may have an unfavorable outcome and may benefit from broader empirical therapy. In turn, IFD increases morbidity, mortality, and the risk of therapeutic failure in patients with HM, in addition to being infections that require lengthy treatment [53]. In this cohort, coexistence of IFD and CRKp-BSI also was associated with poor outcomes.

Despite not being included in the regression model due to its *quasi*-determinant proportionally characteristics, neutropenia must be outlined as a quintessential risk factor. In that direction, strategies to minimize its hazards are under investigation. Granulocyte transfusions are a viable option, and some authors have proposed its use in certain circumstances, such as concomitant IFD and life-threatening BSI, both of which were consistently observed throughout this study [54].

The present study has some limitations, most notably its retrospective observational methodology and single-center small sample size, which might limit the scope and depth of information available. Microbiologic workflow was carried out in the clinical laboratory, using routine protocols throughout the study, and more detailed information regarding mechanisms of resistance and virulence components, as well as clonal evaluation, was not available. Additional work-up was not feasible for all samples. However, the study did find only minor variations in MIC confirmation tests, and illustrated real-life data in which most therapeutic decisions are based on analogous processed microbiological data. It is also important to note that over the long duration of the study, changes in treatment protocols, the availability of new drugs, and improvements in clinical abilities were constantly incorporated, which may have affected the results. Furthermore, this investigation was conducted within a university teaching hospital, where all therapeutic decisions were guided by an established consultative group of infectious disease specialists experienced in managing infections among highly immunocompromised individuals. While statistical approaches could have been used to mitigate these factors, the study was not designed with this in mind, and data from clinical decisions were reported as is. These limitations should be taken into account while interpreting the findings presented in this study.

Among the upcoming therapeutic tools, the introduction of ceftazidime-avibactam may mark a watershed moment in the management of CRKp-BSI. Notwithstanding its limitations, such as high cost and inactivity against some types of carbapenemases–markedly metallo-β-lactamases, but also reports of resistance [55]–it was administered to six patients in this study as part of a combination therapy, and only one died before the 30th day of infection.

Ultimately, this study provided valuable information, reflecting a decade of follow-up in a CRKp endemic area and contributing to filling regional data gaps regarding CRKp-BSI treatment in onco-hematological patients. Even in a resource-constrained setting, the mortality rates were comparable to those previously reported [19–22]. The findings also raise important questions about the value of quinolone prophylaxis and the role of gut colonization surveillance in HM patients and HSCT recipients; they emphasize the prevalence of multiple concurrent serious infections; and the need for tools that allow for the prompt initiation of appropriate antimicrobial therapy. Further, this research embodies observational data in favor of combination therapy for life-threatening infections caused by CR-bacteria, especially in the haematological setting.

## Conclusions

CRKp-BSI in HM patients and HCST recipients was associated with a high mortality rate, particularly in cases of hypotension at presentation and concurrent invasive fungal disease. The early initiation of appropriate treatment, coupled with the use of combination therapy, has the potential to reduce mortality rates, and seems advisable therapeutic goals until more comprehensive data on new drugs and diagnostic tools, particularly those designed for rapid detection of resistance mechanisms, become widely available.

## Supporting information

S1 Fig Gram-negative spectrum antimicrobials used as definitive treatment. The figure illustrates all regimens adopted as definitive treatment for CRKp-BSI during the 10-year span of the study, highlighting the dynamic nature and diversity of therapeutic decisions. All GNB-spectrum antimicrobials used for more than one day were included. The use of a single CRKp active drug throughout the episode was categorized as monotherapy, while combination therapy encompassed the simultaneous use of two or more CRKp active drugs. Treatment was considered inappropriate if it did not include a CRKp active drug. Each line in the figure represents selected antimicrobials used for a single patient. A black square indicates that a drug was used as an active agent, grey squares denote that the drug was part of the definitive therapy despite its in vitro resistance, and white squares correspond to drugs not used in the definitive treatment. Notes: AMK: amikacin; CAV: Ceftazidime-avibactam; CT: combination therapy; GEN: Gentamicin; IN: inappropriate definitive therapy; MER: meropenem; MT: monotherapy; PB: polymyxin B; TIG: tigecycline; FOS: fosfomycin.
(TIF)

S1 File Minimal dataset. Dataset containing information used in the study analysis, for each participant. Original data, in *Portuguese*, was translated to English.
(XLSX)
