# Supplementary figures and images for: Carbapenem-resistant Klebsiella pneumoniae bloodstream infections in haematological malignances and hematopoietic stem cell transplantation: Clinical impact of combination therapy in a 10-year Brazilian cohort
Source: PLoS One. 2024 Jan 26;19(1):e0297161. doi: 10.1371/journal.pone.0297161 (PMC10817138; doi:10.1371/journal.pone.0297161)

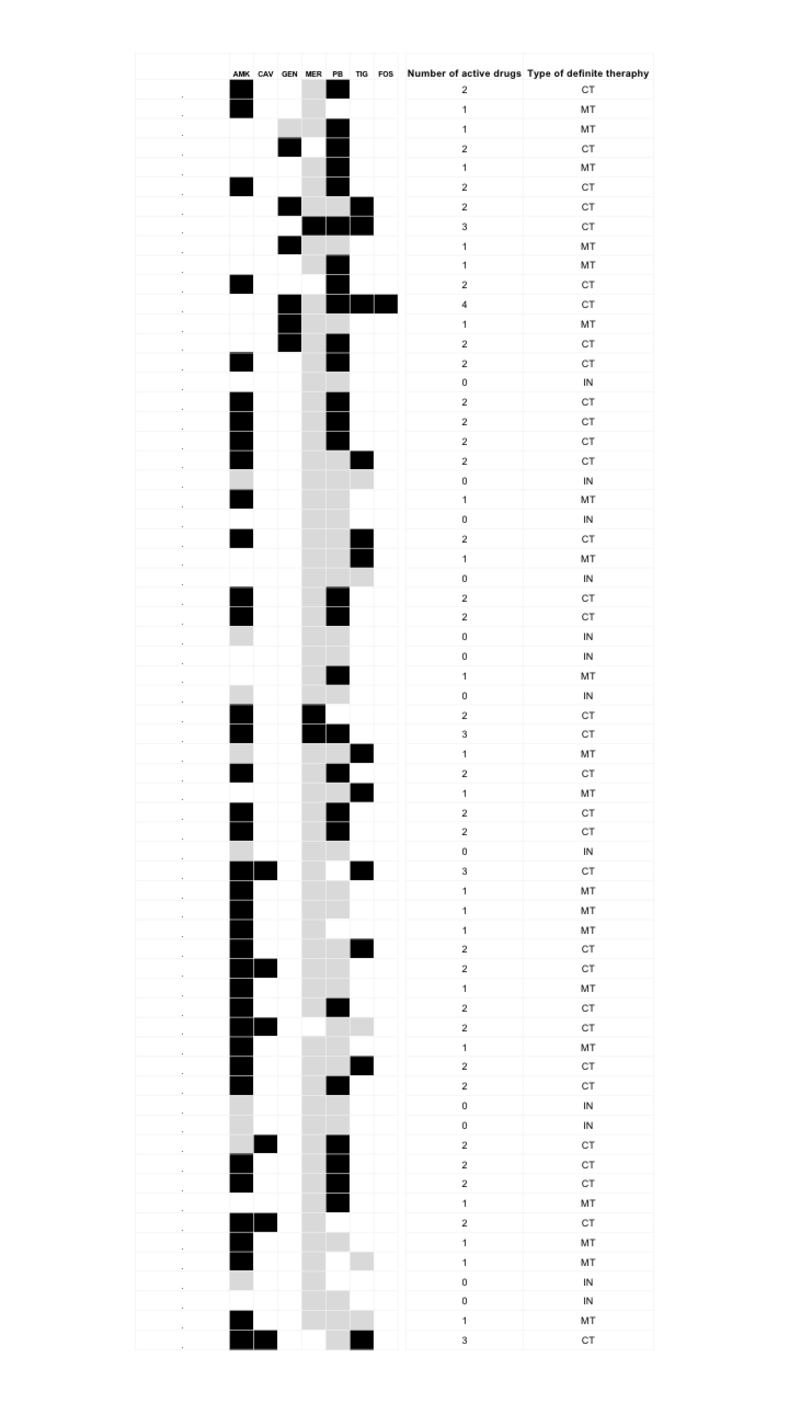

Supplement: S1 Fig — The figure illustrates all regimens adopted as definitive treatment for CRKp-BSI during the 10-year span of the study, highlighting the dynamic nature and diversity of therapeutic decisions. All GNB-spectrum antimicrobials used for more than one day were included. The use of a single CRKp active drug throughout the episode was categorized as monotherapy, while combination therapy encompassed the simultaneous use of two or more CRKp active drugs. Treatment was considered inappropriate if it did not include a CRKp active drug. Each line in the figure represents selected antimicrobials used for a single patient. A black square indicates that a drug was used as an active agent, grey squares denote that the drug was part of the definitive therapy despite its in vitro resistance, and white squares correspond to drugs not used in the definitive treatment. Notes: AMK: amikacin; CAV: Ceftazidime-avibactam; CT: combination therapy; GEN: Gentamicin; IN: inappropriate definitive therapy; MER: meropenem; MT: monotherapy; PB: polymyxin B; TIG: tigecycline; FOS: fosfomycin. (TIF) [file pone.0297161.s001.tif]
